# *N*, *N*-Dimethyl-4-Aminopyridine- and Aluminum Isopropoxide-Catalysed Ring-Opening Polymerizations of *β*-Butyrolactone for the Antimicrobial Oligohydroxybutyrate

**DOI:** 10.3390/ijms27020999

**Published:** 2026-01-19

**Authors:** Qi Bao, Pui-Kin So, Siu Lun Leung, Polly Hang-Mei Leung, Xiaoming Tao

**Affiliations:** 1 Research Institute for Intelligent Wearable Systems, The Hong Kong Polytechnic University, Hong Kong 999077, China; pauki.bao@polyu.edu.hk (Q.B.); polly.hm.leung@polyu.edu.hk (P.H.-M.L.); 2 School of Fashion and Textiles, The Hong Kong Polytechnic University, Hong Kong 999077, China; 3 Research Facility in Life Sciences, The Hong Kong Polytechnic University, Hong Kong 999077, China; pui-kin.so@polyu.edu.hk (P.-K.S.); alan.sl.leung@polyu.edu.hk (S.L.L.); 4 Department of Health Technology and Informatics, The Hong Kong Polytechnic University, Hong Kong 999077, China

**Keywords:** polyhydroxybutyrate (PHB), polyhydroxyalkanoate (PHA), ring-opening polymerization (ROP), *β*-butyrolactone, anionic polymerization catalyst, antimicrobial

## Abstract

Infectious pathogens pose serious threats to public health, necessitating the development of more antimicrobials. In this study, oligohydroxybutyrates were obtained through the catalyzed polymerization of *β*-butyrolactone using *N*, *N*-dimethyl-4-aminopyridine (DMAP) and aluminum isopropoxide [Al(O*i*Pr)_3_] and applied as sustainable antimicrobial agents. The poly3-hydroxybutyrate (PHB) oligomers exhibited broad-spectrum antibacterial activities against both Gram-negative (*E*. *coli*) and Gram-positive (*S*. *aureus*) model bacteria. Additionally, PHB oligomers displayed robust (inhibiting rate: >95%) and rapid (action time: <20 min) antiviral activity against three notorious single-stranded RNA viruses, that is, influenza A virus (H1N1 and H3N2) and coronavirus (SARS-CoV-2). In particular, a comprehensive set of advanced experimental characterizations, including FT-IR, ^1^H- and ^13^C-NMR, and H-ESI-MS/MS, was applied to analyze their chemical structures. The results confirmed the loss of terminal hydroxyl groups in the PHB intermediate and end products associated with theoretical calculations. These findings will also help provide deep insight into the major chain growth mechanism during the synthesis of PHB. The structural variations, which were treated as unwanted side reactions, were identified as a pivotal factor by deactivating the terminal hydroxy during chain growth. Their effective sterilization properties and degradability endowed the as-prepared PHB oligomers with a promising biomedical potential, including for use as disinfectants, sanitizers, and antiseptics.

## 1. Introduction

Polyhydroxyalkanoates (PHAs) are currently recognized as some of the most promising environmentally benign and degradable biopolymers for a wide range of applications [1,2,3]. As the most ubiquitous PHAs, poly3-hydroxybutyrates (PHBs) and their copolymers can be applied in multifarious fields, like textiles, food packaging, and tissue engineering, without extra concerns about pollution [4,5,6]. Meanwhile, PHBs also benefit from their excellent biocompatibility, making them an attractive alternative to some conventional polyolefins derived from petrochemicals [7]. The antibacterial effect of PHBV in knitted polylactide/poly(hydroxybutyrate-co-hydroxyvalerate) (PLA/PHBV) fabrics was first discovered by Huang et al. [8]. Then, the antimicrobial ability and tissue safety of PHBV oligomers extracted from PHBV powders were confirmed by Ma et al. [9]. Afterwards, Zhang et al. discovered the synergistic effects of liquid polyethylene glycol (PEG 200) with synthetic PHB oligomers in terms of their antimicrobial performance [10]. Grażyna Adamus and co-workers pointed out that PHB oligomers did not induce cytoprotective activity, indicating low or no toxicity [11]. Furthermore, PHB oligomers offer another advantage, as they pose no secondary ecological hazards from residual nitrogen*-* and/or phosphorus*-* containing compounds, given that they are composed solely of C, H, and O.

Generally, PHBs originate from two major sources, i.e., microbial fermentation and artificial polymerization. The former mainly involves a series of downstream processes, including extraction, separation, and purification, since PHBs are intracellular energy storage substances within certain microorganisms [12,13,14]. However, this method suffers from several disadvantages, e.g., a time-consuming production period, limited control over reaction conditions, and low yields with high levels of impurities in the final products. Meanwhile, microbially produced PHB granules are highly isotactic, stiff, and brittle, exhibiting high crystallinity and low elongation at break [15]. To address this, artificial synthesis through anionic and/or cationic ring-opening polymerizations (ROPs) using *β*-butyrolactone (BL) as a monomer has been proposed. The processability and thermomechanical properties of synthetic PHB have been improved to some extent [16,17]. Almost 40 years ago, BL monomers were found to be polymerized in an anionic manner in the presence of K in THF containing 18-crown-6 [11]. Afterwards, metal complexes (e.g., Cr [18,19], Ti [20], Sn [21,22], Ln [23], In [24,25], Zr [26], Y [27], and Zn [28,29]) bearing porphyrin/Schiff/Salan ligands were designed and reported as catalysts [29,30,31,32,33,34]. In particular, some low-level-toxicity catalysts, such as Mg complexes, have been found to avoid the potential bio-hazards of metal [35]. The commercially available Al (Ⅲ) isopropoxide [Al(O*i*Pr)_3_] in chlorobenzene has been applied for initializing the polymerization of lactide, as reported by Maciej Bero et al. in 1990 [36]. Kouki Matsubara et al. reported that pyridine (Py) and Al(O*i*Pr)_3_ initiated the ROP of lactide and lactone, yielding block copolymers [37]. In the latter case, Py acted as both the ligand for the Al catalysts and the solvent for the catalysts and monomers. In fact, both solvents and ligand selection/design are crucial in optimizing ROP reactions. James Beament et al. thought that the presence of nucleophilic organic bases acting as ligands could affect the reactivity of the metal centers to lactone monomers and thus facilitate the ROP reaction [38,39]. Nonetheless, Py features high volatility, with unpleasant odors coupled with severe ecotoxicity.

Herein, we report an odorless Py analogue, namely, *N*, *N*-dimethyl-4-aminopyridine (DMAP), that is used to supplant the Py molecule. The basicity and nucleophilicity of DMAP were found to be much stronger than those of Py due to the resonance between the aromatic ring and the pair electrons borne by the nitrogen atom of the dimethylamino group. Meanwhile, DMAP was found to be an efficient organocatalyst for initiating the polymerization of lactide monomers [40,41,42]. In this work, the catalytic system including DMAP and Al(O*i*Pr)_3_ in phenethyl alcohol (PEA) is studied and reported for initiating the ROP of BL for the first time. The use of DMAP was expected to further enhance the polymerization reactions of BL as initiators through electronic and steric effects. The antibacterial activity of PHB oligomers was further validated, addressing some questions regarding their antibacterial effectiveness. Furthermore, the molecular structures of the as-prepared PHB oligomers within this catalyst system were investigated and elucidated thoroughly via advanced techniques along with theoretical calculations. At present, the artificial polymerization of BL to produce PHB comparable to the microbially fermented one in terms of molecular weight remains particularly challenging [11]. Further discussion is given regarding molecular origins, unveiling the root that hinders the continual chain growth of PHB oligomers. Notably, the border functionality of PHB bioactive oligomers, i.e., antiviral activity against influenza virus and coronavirus, is studied and reported for the first time. As macromolecular organic acid-type antimicrobial agents, the antimicrobial properties of PHB oligomers are fully displayed in our pursuit of antimicrobial agents for use in medical fabrics or plastics acting as the surface finishing agents or additives [43].

## 2. Results and Discussion

The performances regarding the catalytic ROP of BL within various catalyst systems are compared and listed in Table 1. No detectable polymerization was observed in the presence of the sole Al(O*i*Pr)_3_ initiator, although it was reported in regard to the catalytic activity observed in the ROP of lactide. Interestingly, Py on its own could initiate the ROP of BL monomer, acting as both a solvent and an organocatalyst beyond a ligand, yet the yield was low (18.3%). By comparison, the DMAP within the PEA system with a 9:1 monomer/catalyst ratio at a temperature of 55 °C displayed better performance in terms of the yield and *M*_n_ of PHB oligomers. The addition of Al(O*i*Pr)_3_ further improved the catalytic ROP performance of the DMAP/PEA system. Meanwhile, solvent effects also played a role in this ROP process since the *M*_n_ (821) obtained from the PEA system was longer than that of the BnH solvent (*M*_n_ = 515).

**Figure 1:**
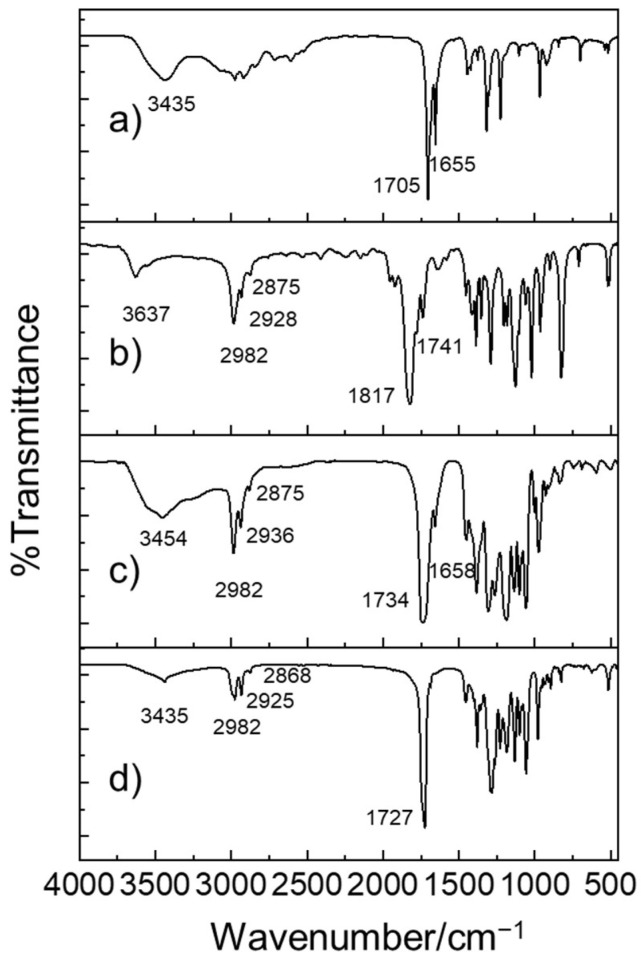
FT-IR transmission spectra of (**a**) *rac-β*-hydroxybutyrate, (**b**) *rac-β-*butyrolactone, (**c**) as-prepared PHB oligomers catalyzed by Al(O*i*Pr)_3_/DMAP, (**d**) as-prepared PHB oligomers catalyzed by Al(O*i*Pr)_3_/DMAP, and bacterially produced PHB polymers.

The structural properties of the as-prepared PHB oligomers were studied via FT-IR. As shown in Figure 1, a comparison of the FT-IR spectra of the BL monomers and as-prepared PHB before and after ROP reactions reveals three distinctive spectral differences in the intensity of the bands: stretching vibrations of hydroxyl groups *v*(O–H), the (anti)symmetric stretching vibration of carboxyl groups *v*(−COO−), and (anti)symmetric C–H vibrations. The typical stretching vibration peaks of the O−H of hydroxyl groups were evident in the range from 3454 cm^−1^ to 3637 cm^−1^. The *v*(O–H) of PHB centered at approximately 3454 cm^−1^ displayed a broad band due to hydrogen bond formation by the OH (sub)groups of carboxyl and hydroxyl groups. In BL monomers, the (anti)symmetric C–H vibrations of primary (–CH_3_), secondary (–CH_2_–), and tertiary (–CH=) alkyl groups were observed in the range from 3000 cm^−1^ to 2875 cm^−1^. Also, (anti)symmetric C=O stretching vibrations of –COO– were observed in the range from 17,050 cm^−1^ to 1817 cm^−1^, indicating the presence of ester bonds. Furthermore, (anti)symmetric C=O stretching vibrations of –COOH or –COO^−^ appeared in the lower-frequency region from 1655 cm^−1^ to 1658 cm^−1^. As the polymerization reaction proceeded, the *v*(O–H) signal and the *v*(C=O) of carboxyl terminal groups became weaker.

**Figure 2:**
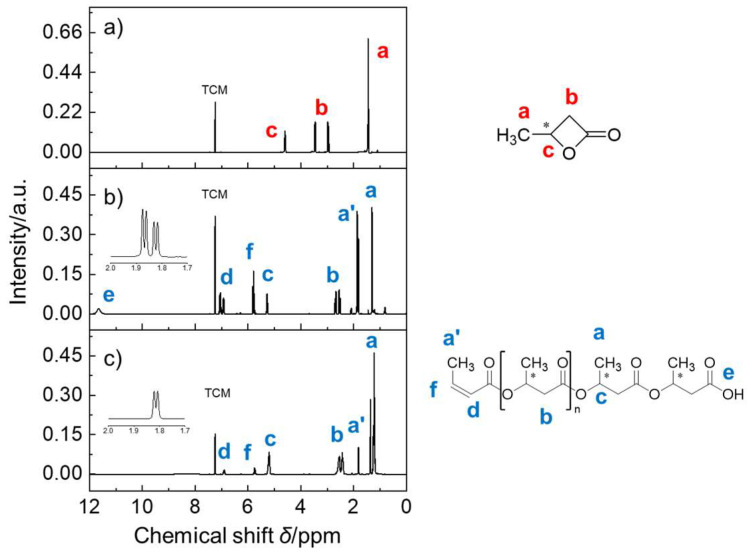
^1^H NMR spectra of (**a**) *rac-β-*butyrolactone, (**b**) as-prepared PHB oligomers catalyzed by Al(O*i*Pr)_3_/Py, and (**c**) as-prepared PHB oligomers catalyzed by Al(O*i*Pr)_3_/DMAP. Inset: Corresponding chemical structure of PHB and enlarged partial spectrum in the range from 1.7 to 2.0 ppm. Note: TCM denotes the internal solvent chloroform.

The as-prepared PHB samples were subjected to NMR analyses. ^1^H NMR analysis revealed three major peaks (a, b, and c) for BL monomers, as shown in Figure 2. The resonance peaks of the BL monomers appeared at around 1.46 ppm (peak a), 2.97~3.45 ppm (peak b), and 4.60 ppm (peak c), a finding in agreement with the peaks corresponding to their molar fractions, namely, methyl CH_3_, methylene CH_2_, and methine CH, respectively. After the ROP reaction, peak b shifted to a higher field location. Meanwhile, the small peak (e) appeared at around 11.7 ppm, indicating the presence of free carboxylic acid groups. Additionally, proton resonances at around *δ* = 5.78 ppm (peak f) and around *δ* = 1.89 ppm (peak a’, d(^3^*J*)) were identified and assigned to the signals of methine CH and methyl CH_3_ of the ethylene (C=C) unit within the as-prepared PHB polymers, as shown in the expanded inset plot. The appearance of the a’ peak (Δ^3^*J*_ab_ = 5 Hz) could be attributed to the formation of the *cis*- and *trans*- isomers as a result of the existence of ethylene terminal groups. Anyway, these side products were identified and formed by the loss of the terminal hydroxy from PHB, as expected.

**Figure 3:**
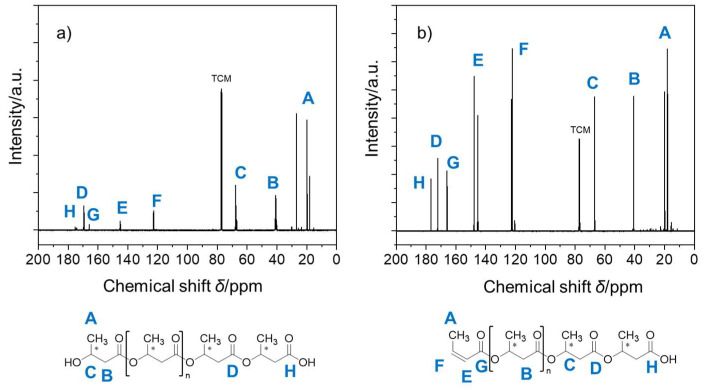
^13^C NMR spectra of (**a**) as-prepared PHB oligomers catalyzed by Al(O*i*Pr)_3_/Py and (**b**) as-prepared PHB oligomers catalyzed by Al(O*i*Pr)_3_/DMAP in CDCl_3_. Note: TCM denotes the internal solvent chloroform.

To further confirm the molecular structures of the PHB polymers, ^13^C NMR analysis was conducted to reveal the peaks for the end group unit in PHB (Figure 3). The ^13^C NMR (298 K, CDCl_3_) spectra showed two clear resonances at around *δ* = 122.7 ppm (peak F) and 145.1 ppm (peak E) for both the PHB moieties. Obviously, such C-13 peaks were due to the unsaturated ethylene groups of the terminal unit, a finding consistent with their proton peaks (peaks d and f) corresponding to the presence of the crotonic acid ester units in PHB oligomers, as described previously. This crotonyl unit was formed through the dehydration reaction as a result of intramolecular *β*-elimination or the *E*1 process of terminal hydroxyl groups together with the adjacent *β*-methylene hydrogen atoms and/or intermolecular *β*-elimination or *E*2 reactions that result in PHB depolymerization [44]. Thereafter, the presence of the ethylene (C=C) unit, along with the loss of the hydroxyl terminal groups after initiation by the catalysts, was identified using NMR measurements.

**Figure 4:**
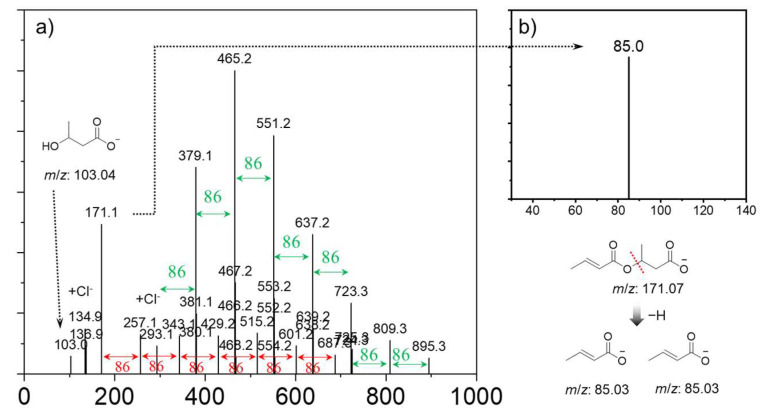
(**a**) Full-scan mass-spectrometry data pertaining to the as-prepared PHB oligomers formed using Al(O*i*Pr)_3_/Py catalyst in the range of 50~1000 amu, with all fragment ions annotated, and (**b**) experimental tandem MS (MS^2^) spectra of targeted fragment ion (*m*/*z* 171.1).

To further identify the PHB products synthesized, Orbitrap tandem mass-spectrometry (H-ESI MS/MS) analysis was carried out. These fragmentation patterns were identified through analysis of the accurate mass-to-charge (*m*/*z*) values of fragment ions obtained via H-ESI MS/MS spectroscopy. As shown in Figure 4a, we found a series of regular signals with a peak-to-peak *m*/*z* increment (Δ*m*/*z*) of 86.0 atomic mass units (amu) between two adjacent signals across the entire spectrum. The two adjacent signals were attributed to a repeating monomer unit of PHB. The most intense peaks in terms of relative abundance in the H-ESI MS/MS spectra were located at *m*/*z* 465.2 [M+Cl]^−^. The average and maximum degrees of polymerization of PHB were *n* = 4 and *n* = 10, respectively. By comparison, the average and maximum degrees of polymerization of the PHB synthesized using Al(O*i*Pr)_3_/DMAP catalyst were larger (*n* = 7~8, *n* = 12), indicating higher efficiency (Figure S1). The MS^2^ fragmentation patterns of the parent ion (*m*/*z* 171.1) provide structurally informative fragment ions with cleavage pathways of the noted C-O chemical bonds (Figure 4b). The crotonic acid anions (*m*/*z* 85.0) was formed due to the cleavage of the polar bond between the *β*-carbon and oxygen atom (alkyl cleavage). In good accordance with the NMR results, H-ESI MS/MS also revealed that the PHB oligomers with crotonyl terminals were formed after the intramolecular and/or intermolecular dehydration during the polymerization of *β*-butyrolactone. However, it should be noted that the thermal stability of PHB oligomers was not high at 300 °C within the ionization source of H-ESI MS/MS, as shown in the TGA data (Figure S2).

**Figure 5:**
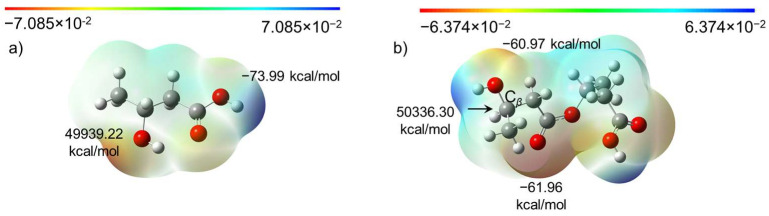
Molecular electrostatic potential maps of (**a**) *β*-hydroxybutyrate and (**b**) PHB dimer oligomers (*n* = 2).

Molecular electrostatic potential (ESP) analysis revealed that the maximum and minimum ESP values of *β*-hydroxybutyrate were located at the O atom of the hydroxyl groups and the H atom of carboxyl groups, as expected, respectively (Figure 5a). However, after polymerization with one more *β*-hydroxybutyrate monomer, the max point relocated to the *β*-C atom of the hydroxyl groups of the dimer that was formed, bearing a positive charge of 50,336.30 kcal/mol (Figure 5b). The secondary minimum negative point was located at the O atom of the hydroxyl groups connected to the *β*-C atom. Such variation will destabilize the molecular structure and undermine polymerization reactivity. The strength of the C-O bond strength became weak, and then terminal hydroxy groups became vulnerable to intermolecular or intramolecular nucleophilic/electrophilic attack, leading to the formation of crotonate groups. This finding is in good agreement with the experimental NMR and H-ESI MS/MS results. In this regard, the polymerization reaction could be anionic polymerization after the loss of terminal hydroxy groups.

**Figure 6:**
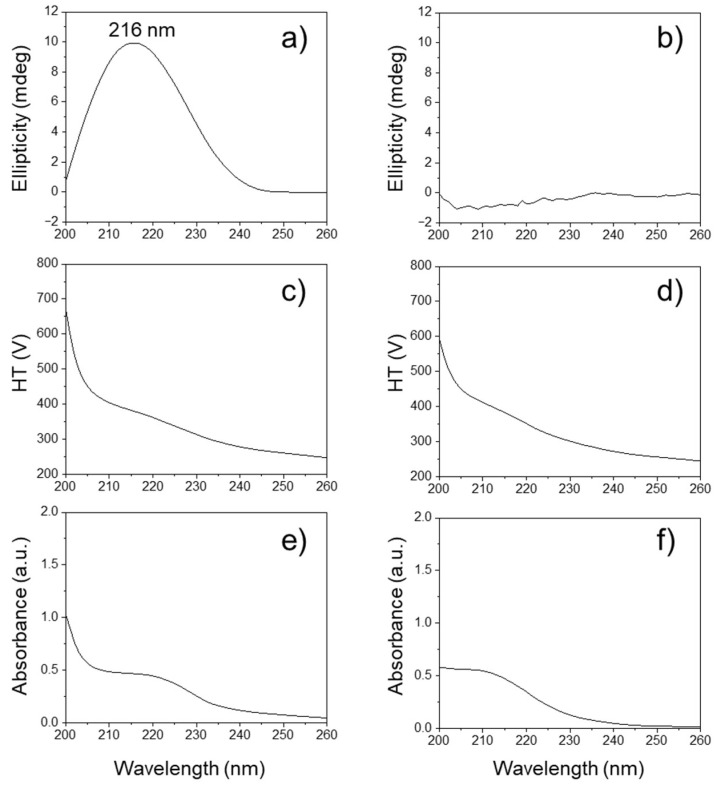
Circular dichroism (CD) spectra of (**a**) bacterially produced PHB oligomers, i.e., Poly[(*R*)-3-hydroxybutyrate], and (**b**) as-prepared racemic PHB oligomers. The high tension (HT) voltage applied to the photomultiplier tube detector for (**c**) bacterially produced PHB oligomers and (**d**) as-prepared racemic PHB oligomers. Ultraviolet (UV) adsorption spectra of (**e**) bacterially produced PHB oligomers and (**f**) as-prepared racemic PHB oligomers.

Circular dichroism (CD) spectroscopy is a powerful tool for analyzing conformational properties, providing deep insights into the structural and chiroptical characteristics of biomolecules [45]. As shown in Figure 6a, the bacterially produced natural PHB oligomers displayed a characteristic broad positive CD band at approximately 216 nm in the ultraviolet wavelength region due to the Cotton effect [46]. This can be interpreted as an indication of the presence of a chiral center in the isotactic oligoester chain of natural poly[(*R*)-3-hydrobutanoic acid]. However, the as-obtained PHB oligomers display no ellipticity in the range between 200 and 260 nm, depending on their random sequence architectures of syndiotactic PᴰᴸHB oligomers, as shown in Figure 6b. The HT (photo-multiplier) voltage signal is presented in the lower panel (Figure 6c,d). Both the HT voltage values remained below 700 V. The PHB oligomers can absorb far-UV light, so less light will reach the detector, resulting in the HT voltage rising at lower wavelengths. Both PHB isomer oligomers showed similar absorption peaks in the range from 200 nm to 240 nm (Figure 6e,f), which were assigned to the *n* → *p* transitions of carbonyl groups. The similar HT and UV absorption profiles for both PHB oligomers confirmed that the bacterial PHB and the as-prepared *racemic* PHB oligomers were present in comparable concentrations. This implies that any differences in their spectral characteristics (like chirality in the CD spectra) are not due to concentration effects but rather inherent structural differences between the two types of PHB oligomers. The UV-CD spectroscopy results also might provide further evidence of the presence of the C=C terminal groups of the as-prepared PHB oligomers in the far-UV range below 210 nm.

**Figure 7:**
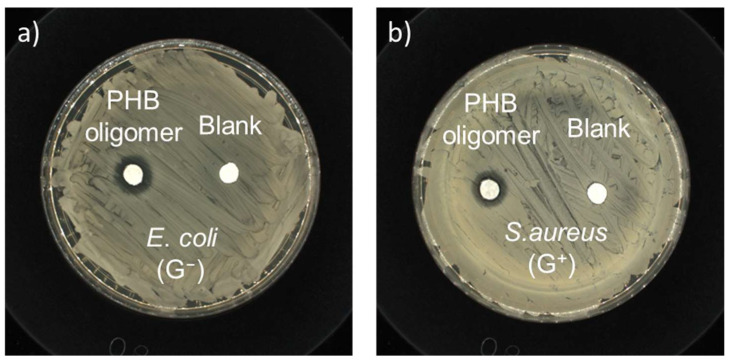
Kirby–Bauer antibiotic strength measurements for the as-prepared-racemic-PHB-oligomer-loaded paper disks and the negative control disk against (**a**) *E*. *coli* (ATCC No. 25922) and (**b**) *S*. *aureus* (ATCC No. 6538).

Antibacterial susceptibility tests were conducted to confirm the antibacterial ability of the PHB oligomers using a typical micro-disk diffusion method (initially). The antibacterial activity of the as-prepared PHB oligomers was compared in the presence of representative Gram-negative (G^−^) bacteria, i.e., *E*. *coli*, and Gram-positive (G^+^) bacteria, i.e., *S*. *aureus*. Visible growth-inhibition rings around the paper disks loaded with PHB oligomers were easily identifiable after incubation with *E*. *coli* and *S*. *aureus* (Figure 7), while no inhibition zone was found around the negative control sample. The inhibitory diameters of the PHB sample were in the range of 1.20~1.25 cm, with a loading density of 0.60 mg/mm^2^. The results confirm qualitatively that both the G^−^ and G^+^ bacterial strains are susceptible to the PHB oligomers.

**Figure 8:**
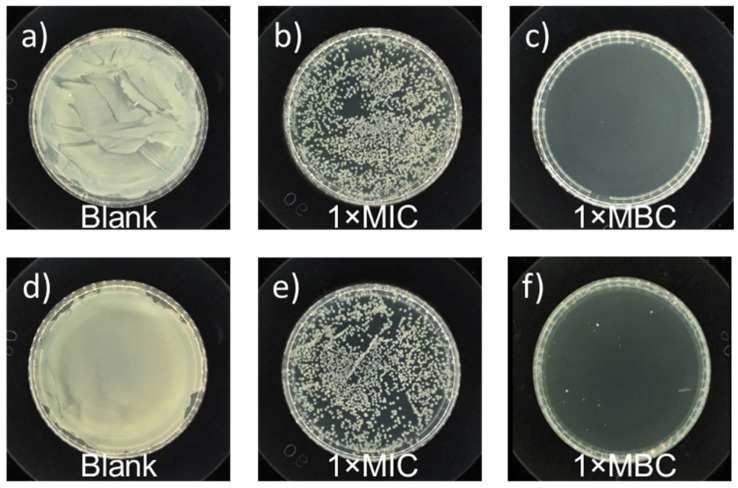
Antibacterial tests conducted against (**a**) *E*. *coli* (ATCC No. 25922) after treatment without and with the as-prepared racemic PHB oligomers at concentrations of (**b**) 1× MIC value and (**c**) 1 × MBC value after 12 h of contact, (**b**) and antibacterial tests conducted against (**d**) *S*. *aureus* (ATCC No. 6538) after treatment without and with the as-prepared racemic PHB oligomers at concentrations of (**e**) 1× MIC value and (**f**) 1× MBC value after 12 h of contact.

To quantitatively investigate the antimicrobial activity of PHB, MIC and MBC values derived via the agar dilution method were applied to determine the antibacterial activity of PHB against *E*. *coli* and *S*. *aureus*. Representative plates were prepared at 37 °C for 12 h using undiluted solutions of the control experiment and samples subjected to PHB treatment, and the results are shown in Figure 8. The control experiments showed robust growth of bacteria. For the PHB-treated samples, all the numbers of living bacterial cells of *E*. *coli* or *S*. *aureus* were greatly reduced. The MIC and MBC values of PHB against *E*. *coli* bacteria were 6.25 mg/mL and 12.5 mg/mL, respectively. The MIC/MBC value of PHB against *S*. *aureus* bacteria was the same. The as-prepared PHB showed broad-spectrum antibacterial properties. Notably, the antibacterial capacity of PHB varied with the concentration of PHB applied, the degree of polymerization, and contact time.

**Figure 9:**
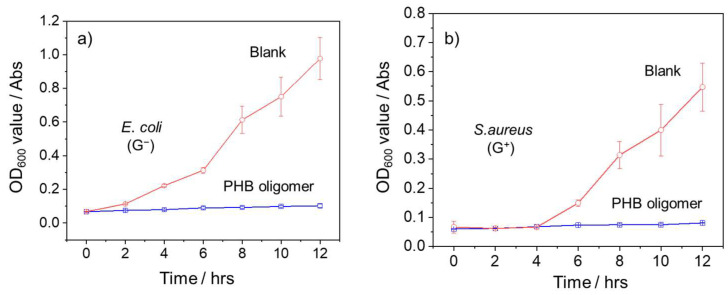
The variation in OD_600_ values in M-H broth containing (**a**) *E*. *coli* (ATCC No. 25922) and (**b**) *S*. *aureus* (ATCC No. 6538) in the presence (blue curve) and absence (red curve) of PHB oligomers (10 mg/mL) provided via treatment conducted at 2 h intervals over 12 h.

To further investigate the antibacterial efficacy of PHB oligomers, a comparative growth kinetics analysis of *E*. *coli* and *S*. *aureus* in the presence and absence of PHB oligomers was also conducted. As depicted in Figure 9, the negative control groups of both bacterial strains experienced rapid logarithmic growth before reaching the stationary stage. In contrast, there was no increase or rebound of the OD_600_ value over 12 h, and no significant bacterial growth was observed when PHB oligomers were present. This result demonstrates that PHB oligomers can completely inhibit the growth of *E*. *coli* and *S*. *aureus* at a concentration of 10 mg mL^−1^.

In recent years, the major threats to public health have mainly been posed by infectious viruses. Consequently, the development of fast and effective antiviral agents has been assigned top priority. The antiviral properties of PHB oligomers were evaluated using Influenza A viruses (IAVs), specifically H1N1 and H3N2, which are known to cause severe respiratory illnesses, serving as model-testing viruses. As shown in Table 2, PHB oligomers offered a rapid (<10 min) and highly effective inhibition rate (>99%) against H1N1 and H3N2 viruses at a concentration of 20 mg/mL. The TCID_50_ value was reduced by three orders of magnitude within 10 min following PHB treatment. Additionally, the PHB oligomers showed good compatibility with MDCK cells at the same concentration. The antiviral effectiveness of PHB oligomers against H1N1 and H3N2 was dependent on both concentration and treatment time. The highest virucidal rate (>99.99%) was achieved 2 h after positive treatment. Compared to the negative control, the TCID_50_ value of the PHB-treated IAV was reduced by at least four orders of magnitude. This indicates that PHB oligomers can effectively neutralize the virus before it spreads, as the influenza virus life cycle ranges from 8 to 10 h. Their antiviral action may result from DNA damage, interruption of metabolic pathways, the production of reactive oxygen species, enzyme/protein deactivation, membrane breakage, etc.

The severe acute respiratory syndrome coronavirus (SARS-CoV) virus and its variants, the chief culprits behind the COVID-19 pandemic, led to over 7.1 million deaths. The antiviral effectiveness of PHB oligomers was tested against the SARS-CoV-2 virus, and the results are also listed in Table 2. Normally, the SARS-CoV-2 virus can survive for 7.96 h to 10.2 h on the surfaces of common stainless-steel/glass/plastic items, a span much longer than that for IVA (1.65~2.00 h) [47]. The virus titer (TCID_50_/mL) value of SARS-CoV-2 was reduced by 1.32 orders of magnitude within 20 min in the presence of 10 mg/mL of PHB oligomers, revealing the rapid antiviral activity of PHB oligomers. No significant improvement in the PHB-oligomer-related antiviral rate against SARS-CoV-2 virus was observed over 1.5 h. The PHB oligomers began to exhibit slight cytotoxicity against Vero E6 cells once the concentrations had been increased to over 20 mg/mL. This might be related to their chirality, since their *S*-3-hydroxybutyrate unit does not exist within natural living organisms. Such an exogenous component will interfere with in vivo carbohydrate energy metabolism within mammal cells. In addition, the lipophilicity (ClogP) of the PHB oligomers was calculated to be 0.463~1.015 (*n* = 3~4), very close to that (~0.650) of phospholipids, which enhances passive permeability across the cell membrane [48,49].

## 3. Materials and Methods

### 3.1. Materials

The starting monomer *rac-β-*butyrolactone (BL, >95.0%) and initiator aluminum isopropoxide (>98.0%) for chemical synthesis were obtained from Tokyo Chemical Industry (TCI) Chemical Co., Ltd. (Jamestown, RI, USA) without any further purification. Poly[(*R*)-3-hydroxybutyrate] (PHB) powder was obtained from TianAn Biologic Materials Co., Ltd., in Ningbo, Zhejiang, China. Pyridine (Py), 4-dimethylaminopyridine (DMAP), and other reagents were all of analytical grade, purchased from Sigma-Aldrich Co. (St. Louis, MO, USA), and used as received. All the ROP reactions were carried out under an inert gas atmosphere using standard Schlenk techniques and/or in an argon-filled glove box where both O_2_ and H_2_O content were less than 0.1 ppm (LABmaster pro, MBraun, Garching, Germany) unless otherwise noted. In brief, BL (12.2 mL) and Py (20 mL)/DMAP (2 g), dissolved in solvent (20 mL), were mixed in a screw-capped thick-wall flask (Synthware Glass, Chongqing, China). Then, 0.3 g of Al(O*i*Pr)_3_ was added, with stirring applied. The reaction mixture was maintained at 55 °C for 12 h. After the mixture was cooled down, diluted HCl solution (2 M, 60 mL) was added to quench the reaction, followed by extraction with dichloromethane/chloroform (60 mL × 3). The non-aqueous liquids were collected and rotary-evaporated to remove the solvents, and then the residue was eluted with dichloromethane/petroleum ether (5:1, *v*:*v*) in a silica gel column to produce a light-white viscous liquid.

### 3.2. Characterization

All NMR spectra were obtained using a JEOL (Tokyo, Japan) ECZ500R 500 MHz Solid-State NMR spectrometer with tetramethylsilane (0.03% TMS *v*/*v*) as the internal standard at room temperature. Two hundred milligrams of each BL/PHB sample was dissolved in 0.6 mL of deuterated chloroform (CDCl_3_, Sigma-Aldrich), which was then transferred into 5 mm NMR tubes. The chemical shifts are expressed in (*δ*) in ppm from the internal standard (CHCl_3_).

The Fourier transform infrared (FT-IR) spectra were recorded on a Spectrum 100 spectrophotometer (Perkin Elmer, Waltham, MA, USA) with KBr pellets. The experimental conditions employed were as follows: a scanning wavenumber ranging from 4000 cm^−1^ to 650 cm^−1^ was used, and 16 scans at 4 cm^−1^ resolution were obtained and averaged. The circular dichroism (CD) measurements were recorded using a JASCO (Tokyo, Japan) J-1500 circular dichroism spectrometer scanning from 200 nm to 260 nm at a rate of 0.5 nm min^−1^ with a 1 cm quartz cell using ethanol as the solvent. All high-resolution accurate-mass tandem mass spectra were recorded on a ThermoFisher Scientific (Waltham, MA, USA) Orbitrap IQ-X system equipped with a heated electrospray ionization (H-ESI MS/MS) interface and operated in negative ionization mode. The ionization voltage, capillary temperature, vaporizer temperature, sheath gas, and auxiliary gas were set to 3.5 kV, 270 °C, 300 °C, 5, and 2 (arbitrary units), respectively. High-purity nitrogen (99.999%) was used for both spray stabilization and for collision-induced dissociation gas. The degree of polymerization was measured using Waters (Milford, MA, USA) 2414 Refractive Index Detector gel permeation chromatography with polystyrene as a standard.

Computational Methodology: The quantum chemistry calculations were performed using ORCA and the Multiwfn program on a Windows platform, consisting of an Intel Xeon e5266 processor (2.6 GHz 10 Core CPU) with 64 GB DDR4 memory [50,51,52,53]. Molecular geometry optimizations were carried out based on the B3LYP/6-311G level basis set using the DFT-D3 (BJ) dispersion correction.

### 3.3. Antimicrobial Activity Evaluation

The Gram-negative and the Gram-positive bacteria were tested using *Escherichia coli* (*E*. *coli*) ATCC 25922 and *Staphylococcus aureus* (*S*. *aureus*) ATCC 6538 as the model strains, respectively. The antimicrobial susceptibility (disk diffusion) tests in Muller–Hinton (M–H) agar plates were carried out as described previously [47,54]. In brief, a fixed number of bacteria suspensions (10^6^~10^7^ CFU/mL) were spread onto the M–H agar plates with sterile cotton swabs. The PHB-containing and negative control disk samples were placed at the center of each agar plate and pressed gently together. After incubating at 37 °C for 48 h, the agar plates were observed to note the inhibitory zones.

The antibacterial activity of PHB oligomer in terms of the minimum inhibitory concentration (MIC) and the minimum bactericidal concentration (MBC) were also assessed based on the dynamic shake flask contact method. Briefly, a series of PHB oligomers were added to the bacteria containing phosphate-buffered saline (PBS) media (1.5~3.0 × 10^5^ CFU mL^−1^). Notably, a suitable amount of PEG 200 was added to help dissolve the PHB oligomers in the bacterial suspensions. Then, these media were incubated for 12 h under culture conditions of 37 °C and 150 rpm agitation. A bacterial culture incubated with blank PBS instead of PHB served as the negative control. After incubation, the bacterial cultures were diluted serially to 10^3^~10^1^ CFU mL^−1^, and 100 μL of the sample was withdrawn from each diluted solution and spread over the solid agar plates. The solid agar plates were cultivated at 37 °C for 12~48 h. The MIC and MBC values were then obtained using the agar-plate-counting method.

The growth kinetics of bacteria treated with and without PHB oligomers were also detected and compared using a standard broth microdilution method. Briefly, 100 μL of the bacterial culture media (~10^6^ CFU mL^−1^) was transferred into a 96-well plate, and then 100 μL of PHB oligomers with a final concentration of 10 mg/mL was also added and mixed. Bacterial growth was monitored according to OD_600nm_ values in 2 h intervals on a microplate reader at 37 °C.

The virucidal effect of PHB oligomers was investigated in vitro using the virus titer method, following the technical standard for disinfection (2.1.1.10.7) issued by the Ministry of Health, P.R. China (2002) as reported previously [47]. Briefly, Madin–Darby Canine Kidney (MDCK) cells and Vero E6 cells were selected as host cells for *influenza A* virus (IAV) strains H1N1 and H3N2 and *coronavirus* (CoV) strain SARS-CoV-2, respectively. Both MDCK and Vero E6 cells were cultured in Dulbecco’s modified Eagle’s medium (DMEM) supplemented with 10% fetal bovine serum, penicillin (100 U/mL), and streptomycin (100 μg/mL). First, the MDCK/Vero E6 host cells were trypsinized to obtain a single-cell suspension (2 × 10^5^ cells/mL). A total of 100 μL of this cell suspension was added into the 96-well plate. Afterwards, the virus suspensions were serially diluted from 10^−1^ to 10^−7^ in sterile tubes. A total of 100 μL of each viral dilution was also added into the cells in ten replicates and mixed. Every procedure was repeated at least three times under identical conditions. These 96-well plates were transferred into an incubator at 37 °C with 5% CO_2_. Subsequently, an equal volume of PHB oligomer solution was mixed with the viruses (H1N1, H3N2, and SARS-CoV-2) containing media for different time intervals to obtain the experimental group. The negative control was established by mixing the virus with an equal volume of PBS and incubating it for the same time intervals. The cell number showing cytopathic effects (CPE) was recorded to calculate the titer values. Finally, virus titers (TCID_50_) for control and experimental groups were determined, wherein TCID_50_ was defined as the dilution that produced CPE in 50% of the inoculated cell culture wells [55,56].

Results are presented as both percent reductions (%) of the TCID_50_/mL values and Log_10_ TCID_50_/mL reductions.

Then, the virucidal rate was calculated using the following equation:

(1) $$Antiviral\;rate\left(\frac{{TCID}_{50}}{mL}\right)=\frac{{N_{control}-N}_{experimental}}{N_{control}}\times 100\%$$

$${Log}_{10}virus\;infectivity\;titer\;reduction={Log}_{10}{\;N}_{control}-{Log}_{10}N_{experimental}\;$$

where *N*_control_ and *N*_experimental_ correspond to the average TCID_50_/mL values of the control group and the experimental group, respectively.

## 4. Conclusions

To summarize, based on the above results and discussion, tentative conclusions were drawn and discussed, as shown below:

1. DMAP and Al(O*i*Pr)_3_ were employed to improve the ROP reaction pertaining to PHB. DMAP displayed more advantages over pyridine in terms of catalytic efficiency and polymer degree, and it could also initiate the polymerization of BL alone.
2. The FT-IR, ^1^H and ^13^C NMR, and H-ESI MS/MS characterization associated with chemistry-computing calculations revealed the loss of terminal hydroxyl groups of the PHB oligomers.
3. The as-prepared PHB oligomers showed rapid and broad-spectrum antimicrobial effects against a wide range of microbes, ranging from viruses, including influenza A virus (H1N1 and H3N2) and SARS-CoV-2 (COVID-19), to bacteria, including *E*. *coli* and *S*. *aureus*.
4. Furthermore, leveraging the side products of ROP with crotonate end-capping groups endowed PHB with more opportunities via end-chain functionalization in the post-copolymerization process.

## Figures and Tables

**Table 1:** Summary of anionic polymerization of BL in the presence of different catalyst systems.

Catalytic Systems	Conc. [i]_0_ (mol/L) ^1^	Conc. [M]_0_ (mol/L)	Solvent	Temp. ^2^ (°C)	Time (h)	*M*n (Da)	Yield (%)
Al(O*i*Pr)_3_/DMAP	0.073/0.82	7.48	BnH ^3^	55	24	515	78.6
Al(O*i*Pr)_3_/Py	0.073/0.82	7.48	BnH	55	24	465	75.2
Py	12.43	7.48	Py ^4^	55	24	625	18.3
DMAP	0.82	7.48	PEA ^5^	55	24	712	78.4
Al(O*i*Pr)_3_	0.073	7.48	PEA	55	24	—	—
Al(O*i*Pr)_3_/Py	0.073/0.82	7.48	PEA	55	24	730	78.5
Al(O*i*Pr)_3_/DMAP	0.073/0.82	7.48	PEA	55	24	821	85.3

^1^ Conc.: concentration; ^2^ Temp.: temperature; ^3^ BnH: toluene; ^4^ Py: pyridine; ^5^ PEA: phenethyl alcohol.

**Table 2:** Antiviral performance of PHB oligomers against *Influenza A* H1N1, H3N2, and *Coronaviridae* SARS-CoV-2.

Virus Species Tested	Host Cells	Applied Concentration	Contact/Action Time	Average Lg TCID_50_/mL	Logarithm Reduction Value (KL)	Virus Inaction Ratio (%)
H1N1 (ATCC VR-1469)	MDCK	0 mg/mL	10 min	5.58 ± 0.08	—	—
		20 mg/mL	10 min	2.44 ± 0.12	3.14	
		0 mg/mL	2 h	5.71 ± 0.07	—	—
		10 mg/mL	2 h	2.42 ± 0.01	3.30	99.95
		0 mg/mL	2 h	5.74 ± 0.08	—	—
		20 mg/mL	2 h	<1.50	>4.24	>99.99
H3N2 (ATCC VR-1679)	MDCK	0 mg/mL	10 min	5.76 ± 0.07	—	—
		20 mg/mL	10 min	2.45 ± 0.05	3.14	
		0 mg/mL	2 h	5.87 ± 0.06	—	—
		10 mg/mL	2 h	2.44 ± 0.12	3.53	99.97
		0 mg/mL	2 h	5.90 ± 0.10	—	—
		20 mg/mL	2 h	<1.50	>4.40	>99.99
SARS-CoV-2 (IVCAS 6.7585)	Vero E6	0 mg/mL	20 min	7.18 ± 0.14	—	—
		10 mg/mL	20 min	5.50 ± 0.01	1.32	95.16
		0 mg/mL	1.5 h	6.63 ± 0.13	—	—
		10 mg/mL	1.5 h	5.50 ± 0.02	1.13	92.57
		20 mg/mL	1.5 h	5.50 ± 0.05	1.13	92.57

## Data Availability

The original contributions presented in this study are included in the article/Supplementary Materials. Further inquiries can be directed to the corresponding author.

## Supplementary Materials

The supporting information can be downloaded at https://www.mdpi.com/article/10.3390/ijms27020999/s1.
