# Supplementary material for: N, N-Dimethyl-4-Aminopyridine- and Aluminum Isopropoxide-Catalysed Ring-Opening Polymerizations of β-Butyrolactone for the Antimicrobial Oligohydroxybutyrate
Source: Int J Mol Sci. 2026 Jan 19;27(2):999. doi: 10.3390/ijms27020999 (PMC12842008; doi:10.3390/ijms27020999)
Supplement: Supplementary file 1 [file ijms-27-00999-s001.zip › ijms-4083656-supplementary.pptx]

## Slide 1
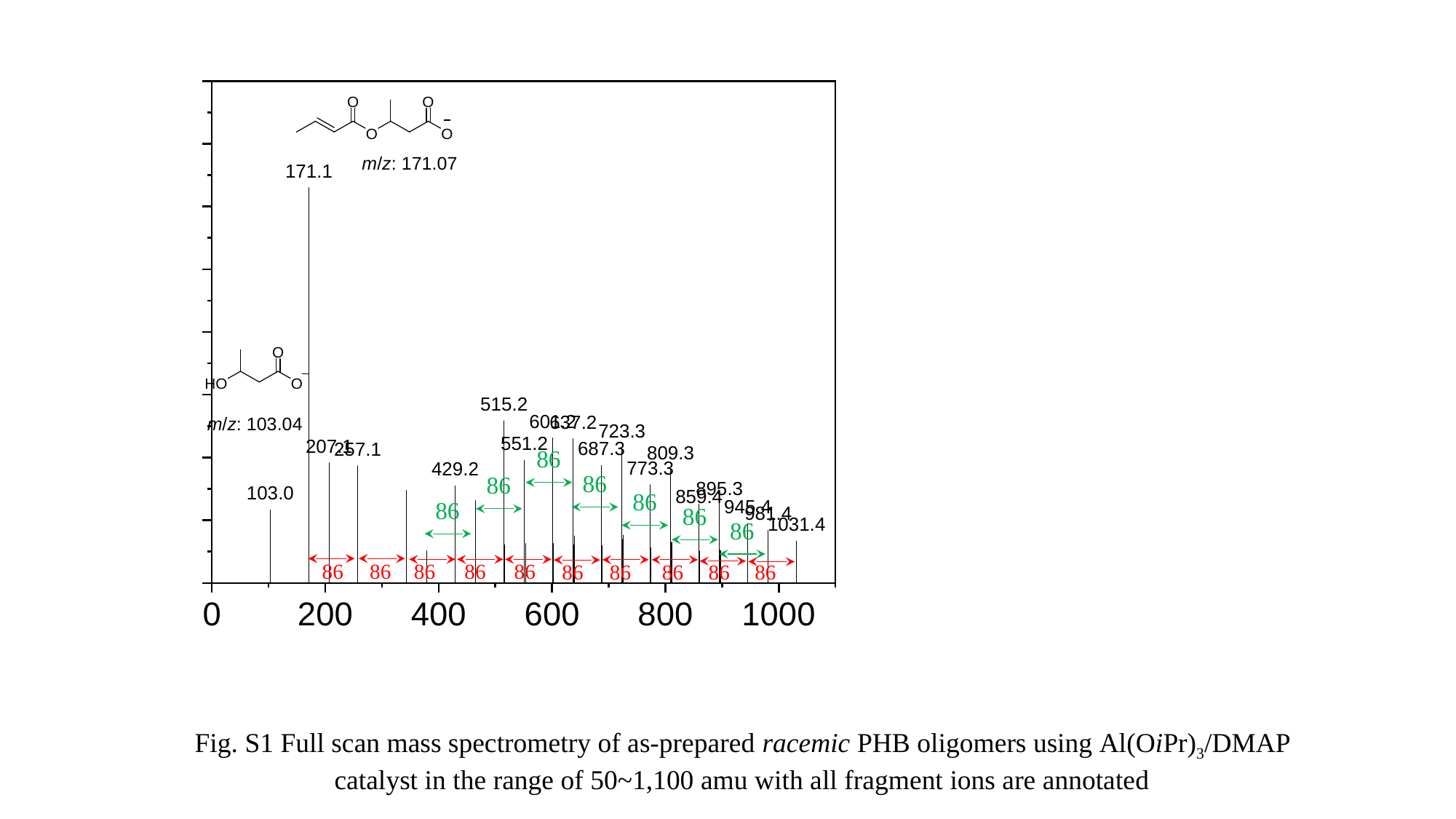

m/z: 171.07
m/z: 103.04
86
86
86
86
86
86
86
86
86
86
86
86
86
86
86
86
86
Fig. S1 Full scan mass spectrometry of as-prepared racemic PHB oligomers using Al(OiPr)3/DMAP catalyst in the range of 50~1,100 amu with all fragment ions are annotated

## Slide 2
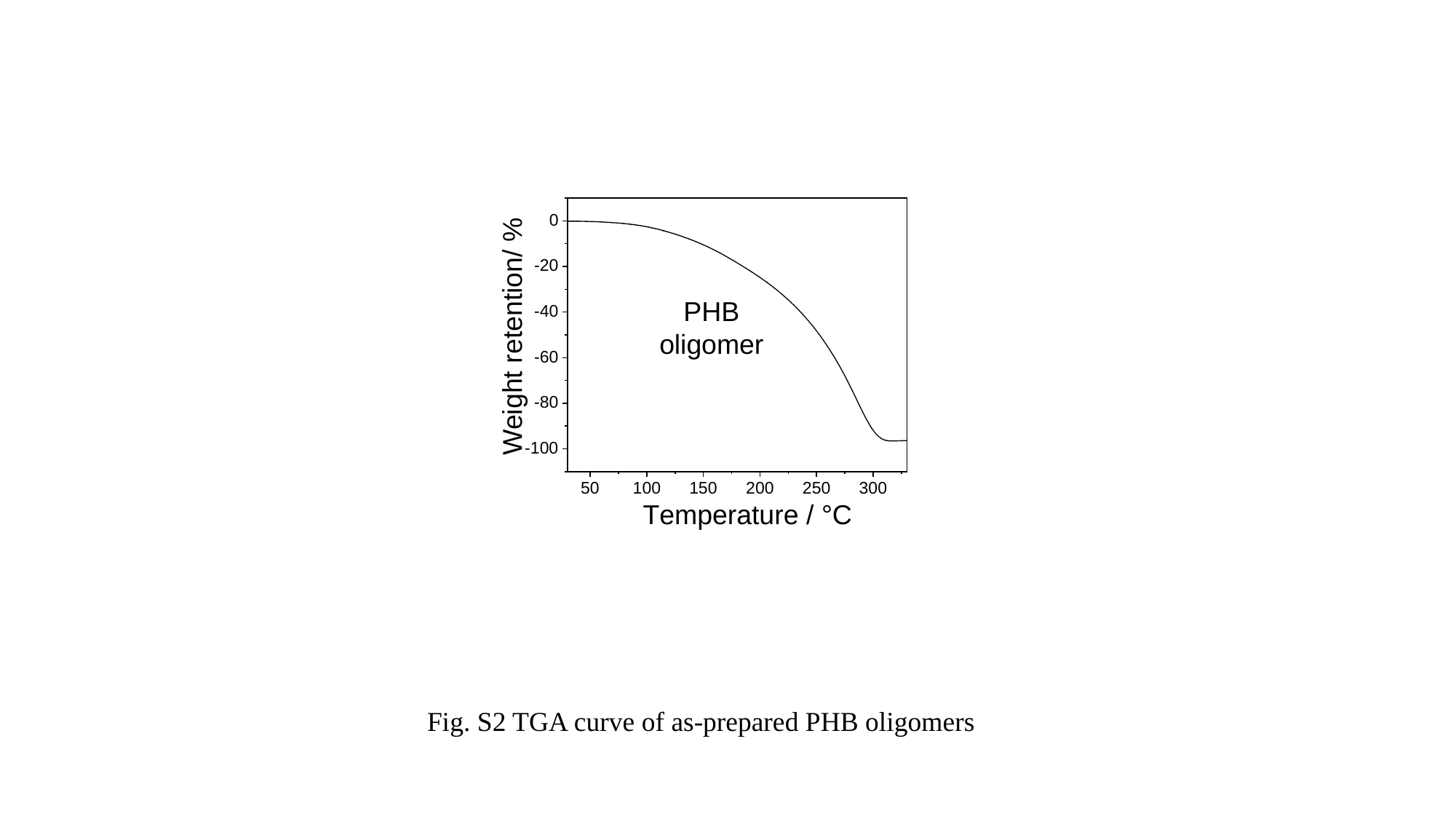

PHB oligomer
Weight retention/ %
Temperature / °C
Fig. S2 TGA curve of as-prepared PHB oligomers
